# Immuno-PET imaging based radioimmunotherapy in head and neck squamous cell carcinoma model

**DOI:** 10.18632/oncotarget.20760

**Published:** 2017-09-08

**Authors:** In Ho Song, Youn Noh, Junhye Kwon, Jae Ho Jung, Byung Chul Lee, Kwang Il Kim, Yong Jin Lee, Joo Hyun Kang, Chae Seo Rhee, Chul Hee Lee, Tae Sup Lee, Ik Joon Choi

**Affiliations:** ^1^ Division of RI Convergence Research, Research Institute of Radiological and Medical Sciences (RIRAMS), Korea Institute of Radiological and Medical Sciences (KIRAMS), Seoul, Republic of Korea; ^2^ Division of Radiological and Clinical Research, Korea Cancer Center Hospital (KCCH), Korea Institute of Radiological and Medical Sciences (KIRAMS), Seoul, Republic of Korea; ^3^ Department of Nuclear Medicine, Seoul National University Bundang Hospital, Seoul National University College of Medicine, Seongnam, Republic of Korea; ^4^ Department of Otorhinolaryngology-Head and Neck Surgery, Seoul National University Bundang Hospital, Seoul National University College of Medicine, Seongnam, Republic of Korea; ^5^ Department of Otorhinolaryngology-Head and Neck Surgery, Korea Cancer Center Hospital (KCCH), Korea Institute of Radiological and Medical Sciences (KIRAMS), Seoul, Republic of Korea

**Keywords:** immuno-PET, radioimmunotherapy, cetuximab, EGFR, head and neck squamous cell carcinoma

## Abstract

The epidermal growth factor receptor (EGFR) is one of the most comprehensively studied molecular targets in head and neck squamous cell carcinoma (HNSCC). However, inherent and acquired resistance are serious problems and are responsible for limited clinical efficacy and tumor recurrence. In this study, we evaluated the feasibility of immuno-positron emission tomography (PET) imaging and radioimmunotherapy (RIT) with ^64^Cu-/^177^Lu-PCTA-cetuximab in cetuximab-resistant SNU-1066 HNSCC xenografted model. The cellular uptake of ^64^Cu/^177^Lu-3,6,9,15-tetraazabicyclo[9.3.1]-pentadeca-1(15),11,13-triene-3,6,9,-triacetic acid (PCTA)-cetuximab showed good correlation with western blot and flow cytometry analysis in EGFR expression level of various HNSCC cells. ^177^Lu-PCTA-cetuximab selectively killed cetuximab-resistant SNU-1066 cells *in vitro*. ^64^Cu-/^177^Lu-PCTA-cetuximab specifically accumulated in SNU-1066 tumor and those uptakes were peaked at 48 h and 7 day, respectively in biodistribution, PET and single-photon emission computed tomography/computed tomography (SPECT/CT) imaging. RIT with single dose of ^177^Lu-PCTA-cetuximab exhibited significant tumor regression and markedly reduced 2-[^18^F]fluoro-2-deoxy-D-glucose (^18^F-FDG) uptake, compared to other groups. Proliferation index were dramatically decreased and apoptotic index increased in RIT group. These results suggest that a diagnostic and therapeutic convergence radiopharmaceutical, ^64^Cu-/^177^Lu-PCTA-cetuximab has the potential of target selection using immuno-PET imaging and targeted therapy by RIT in EGFR expressing cetuximab-resistant HNSCC tumors.

## INTRODUCTION

Head and neck squamous cell carcinoma (HNSCC) is the seventh most common cancer, with an annual worldwide incidence rate of more than 600,000 [1]. In only 50% of HNSCC patients, the current conventional treatment strategies including surgery, chemotherapy, and radiation, are effective. The concomitant chemo-radiation strategy has been used for locally advanced resectable cancers, although there seems to be no survival benefit compared with other treatments [2, 3]. In HNSCC patients with unresectable advanced disease, the combined therapy only achieved suboptimal disease control with a 5-year survival rate of < 10% [4]. Therefore, there is an urgent need for the development of new therapeutic regimens that improve clinical outcome and have better toxicity profile.

As our understanding of the molecular biology of HNSCC continues to improve, this may provide the opportunity to develop molecular targeted therapy for HNSCC treatment. Epidermal growth factor receptor (EGFR) is the most well-studied target for HNSCC and is overexpressed in more than 90% of HNSCC [5–7]. EGFR overexpression is associated with poor prognosis, increased tumor growth, metastasis, and resistance to chemotherapy and radiation therapy [8]. In the past decades, the EGFR has been one of the most comprehensively studied molecular targets in oncology therapeutics. Despite the enthusiasm and optimism accompanying the development of agents targeting EGFR, only a minority of patients have benefited from these drugs. As a monotherapy, small-molecule tyrosine kinase inhibitors and monoclonal antibodies against EGFR, cetuximab and panitumumab, etc, have shown limited efficacy in head and neck cancer [9–12].

Cetuximab, a chimeric anti-EGFR IgG1 is approved by the FDA for HNSCC patients, which binds to EGFR with high affinity, prevents the activation of downstream signaling pathways and also induces antibody-dependent cellular toxicity [13–15]. Nevertheless, one main challenge in the targeted therapy of HNSCC remains, namely intrinsic and acquired drug resistance. Many HNSCC tumors remain unresponsive to EGFR targeted therapy, as for instance low response rate with cetuximab as a single agent [16]. Therefore, alternative therapeutic approach is needed to overcome drug resistance as well as limited response rate in HNSCC patients. Although EGFR inhibition by various inhibitors may not be curative in solid tumors, combination approach with radiotherapy might moderately improve local tumor control [17, 18]. Recently, alternative approach using radiolabeled anti-EGFR antibodies has shown precise quantification of target expression as an immuno-positron emission tomography (PET) agent and enhanced therapeutic efficacy as a radioimmunotherapeutic agent compared to immunotherapy in preclinical HNSCC xenograft models [19, 20]. Cetuximab has been coupled with different radionuclides for imaging and/or treatment of various tumor models for individualized treatment strategies [21].

Immuno-PET imaging is a noninvasive, quantitative whole body imaging strategy for obtaining comprehensive information to target molecules, as opposed to immunohistochemistry in single biopsy. Radioimmunotherapy (RIT) can directly deliver the therapeutic radiation to the tumor site by labeling antibodies with suitable radionuclides [22]. RIT with beta-particle emitter has been proposed in cancers that do not respond to conventional treatments, in particular small solid tumors and could overcome tumoral heterogeneity by “cross-fire” effect. Therapeutic efficacy of RIT solely depend on the radiation dose delivered to target expressing tumor tissues. Immuno-PET has potentials for providing the rationale of antibody targeting against cancer specific targets and dosimetric determinations before RIT. Therapeutic response by RIT could be also predicted by determining tumor uptake and dosimetry of the RIT agent through immuno-PET imaging.

Currently, ^64^Cu and ^89^Zr have been widely used for immuno-PET imaging. ^89^Zr has long half-life (78.4 h) comparable to physiological half-life of whole antibody, but ^89^Zr-labeled antibody showed higher radiation dose for immuno-PET imaging than ^64^Cu-labeled antibody [23]. In radioimmunotherapy, ^90^Y and ^177^Lu have been the most frequently used radionuclides Especially, ^177^Lu is suitable for therapeutic purposes because of the low tissue penetration range with low-energy β^−^-emission (497 keV). ^177^Lu is found to be effective in localizing cytotoxic radiation in relatively small areas and proficient in destroying small tumors as well as metastatic lesions with less damage to surrounding normal tissue and it is particularly well suited for the radiolabeling of antibodies that have slow targeting kinetics [24].

In our recent study, we made diagnostic and therapeutic convergence radiopharmaceutical, ^64^Cu-/^177^Lu-cetuximab for imaging and therapy in EGFR expressing esophageal squamous cell carcinoma (ESCC) model [25]. Immuno-PET imaging represented the extent of target expression and RIT showed marked inhibition of tumor growth in EGFR expressing ESCC. In this study, we aimed to evaluate the feasibility of immuno-PET imaging-based radioimmunotherapy and the therapeutic efficacy with the diagnostic and therapeutic convergence radiopharmaceutical, ^64^Cu-/^177^Lu-cetuximab, in SNU-1066 HNSCC xenograft model, which was resistant to immunotherapy with cetuximab.

## RESULTS

### EGFR expression level and cytotoxicity by cetuximab in HNSCC cell lines

**Figure 1:**
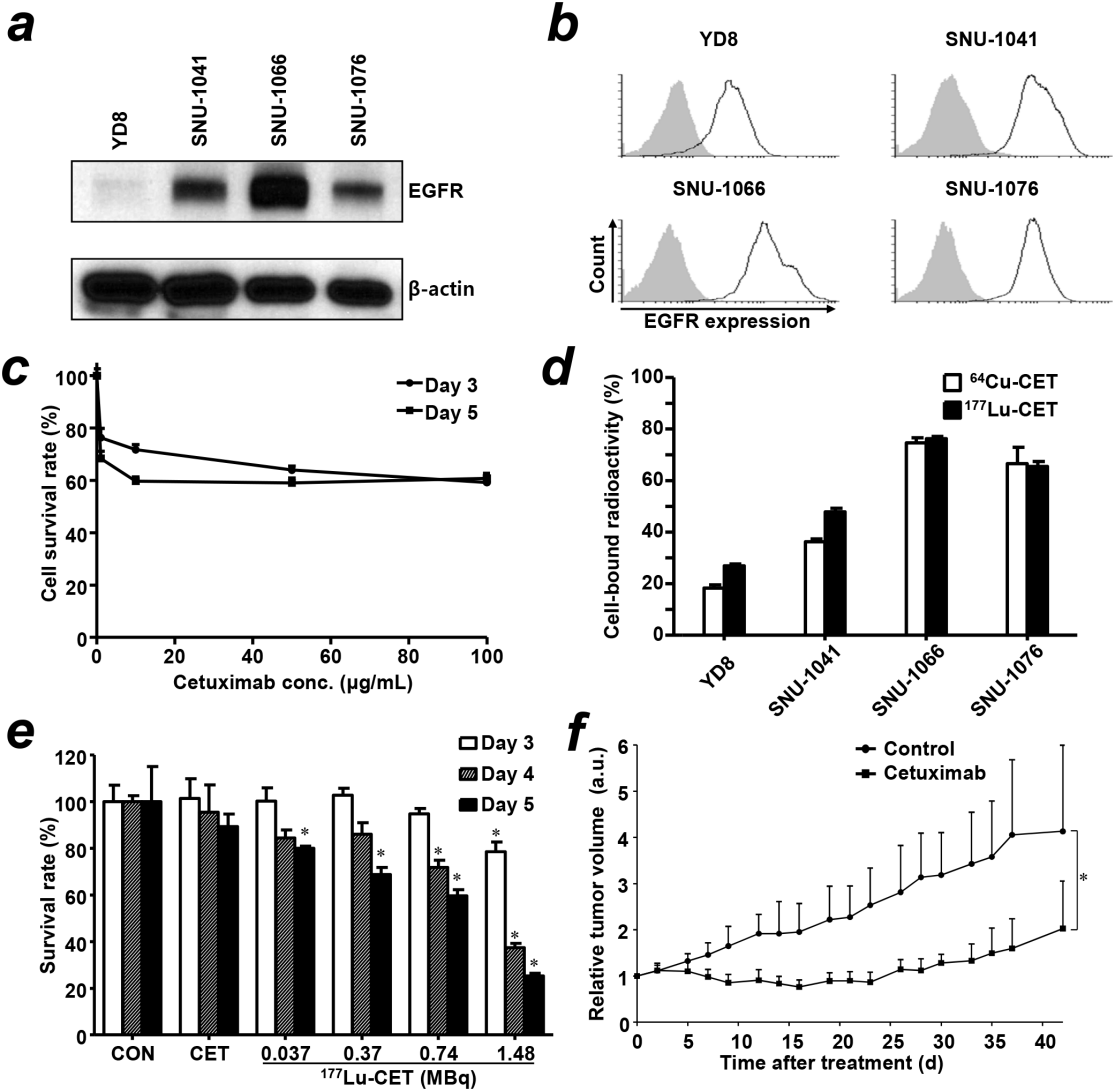
Characterization of EGFR expression in head and neck squamous cell carcinoma (HNSCC) cells, treatment effect of cetuximab and *in vitro* cell binding assay of radiolabeled cetuximab **(a)** Expression level of EGFR protein in HNSCC cells by western blot. **(b)** Flow cytometry in HNSCC cells with cetuximab antibody. Gray shaded curve, isotype control; black lined curve, cetuximab. **(c)** The cytotoxic effect of cetuximab in SNU-1066 HNSCC cells exposed to different concentrations of cetuximab. The viability of SNU1066 cells was evaluated by the MTS assay. **(d)** *In vitro* cell binding assay of ^64^Cu-PCTA-cetuximab (^64^Cu-CET) and ^177^Lu-PCTA-cetuximab (^177^Lu-CET) in HNSCC cells. **(e)** *In vitro* therapeutic efficacy of ^177^Lu-PCTA-cetuximab in SNU-1066 cells. The survival rate (%) of SNU1066 cells was evaluated by Accustain solution (DigitalBio). CON; control, CET; cetuximab. ^*^, vs. control, *P* < 0.05 **(f)** Therapeutic efficacy of cetuximab in SNU-1066 HNSCC xenograft model. The relative tumor volumes were measured after injection of saline (control) and six doses of cetuximab (10 mg/kg, thrice per week for 2 weeks). ^*^, vs. control, *P* <0.05; a.u., arbitrary unit.

The relative expression levels of EGFR in four HNSCC cell lines were determined by western blot (Figure 1a) and flow cytometry (Figure 1b). Median fluorescence intensity was 33.4, 97.3, 112.4, and 91.4 in YD-8, SNU-1041, SNU-1066 and SNU-1076 cells, respectively. As determined by flow cytometry and western blot, SNU-1066 cells expressed relatively higher level of EGFR expression than other HNSCC cells.

The anti-growth effect of cetuximab was determined in SNU-1066 cells exposed to different concentration for day 3 and 5 (Figure 1c). The survival rate (%) of SNU-1066 cells decreased in a dose-dependent manner at below 10 μg/mL. However, the viability of SNU-1066 cells at above 10 μg/mL of cetuximab maintained above 60% at both 3 and 5 day incubation. We could not obtain IC50 value by cetuximab treatment in SNU-1066 cells.

### Characteristics of ^64^Cu- or ^177^Lu-PCTA-cetuximab

The average number of chelates per cetuximab was determined to be 4.0 ± 0.4 by MALDI mass spectrometry. ^64^Cu-/^177^Lu-PCTA-cetxuximab were prepared successfully at high radiolabeling yield (> 98%) and radiochemical purity (> 98%) which were checked by ITLC-sg and size-exclusion HPLC analysis. ^64^Cu- and ^177^Lu-PCTA-cetxuximab have favorable immunreactive index as 0.972 and 0.976, respectively. These radioimmunoconjugate showed good *in vitro* serum stability (above 90%) [25].

To evaluate and compare the EGFR expression level among western blot, flow cytometry and cell binding assay, we performed cell binding assay using ^64^Cu- and ^177^Lu-PCTA-cetuximab (Figure 1d). Cell-bound radioactivities (%) of ^64^Cu-PCTA-cetuximab in HNSCC cell lines were 18.3 ± 1.2% in YD-8, 36.2 ± 1.1% in SNU-1041, 74.6 ± 2.0% in SNU-1066 and 66.5 ± 6.3% in SNU-1076. The cell-bound radioactivities (%) of ^177^Lu-PCTA-cetuximab showed a similar pattern with those of ^64^Cu-PCTA-cetuximab. The cellular binding of radioimmunoconjugates in HNSCC cells was well correlated with the EGFR expression level evaluated by western blot and flow cytometry analysis.

### Cytotoxicity of ^177^Lu-PCTA-cetuximab

To determine the survival rate (%) by increasing radioactivity dose of ^177^Lu-PCTA-cetuximab, SNU-1066 cells were treated with cetuximab (2 μg/mL) and various radiation dose of ^177^Lu-PCTA-cetuximab for 5 days (Figure 1e). There was no cytotoxic effect in cetuximab treated SNU-1066 cells. However, in the different radioactivity dose of ^177^Lu-PCTA-cetuximab-treated cells with same antibody concentration, the survival rate (%) was decreased as a radioactivity dose dependent manner and markedly decreased to 25.3 ± 1.2% at 1.48 MBq dose for 5 day incubation (*P* < 0.001). The cytotoxicity of ^177^Lu-PCTA-cetuximab also increased as incubation time-dependent manner. These results suggest that beta irradiation from ^177^Lu-PCTA-cetuximab could effectively kill in EGFR expressing and cetuximab-resistant HNSCC cells as a radiation dose dependent manner.

### Immunotherapy

Therapeutic effect of cetuximab in SNU-1066 HNSCC xenograft model was represented Figure 1f. Cetuximab showed slight inhibition in tumor growth during i.v. injection of six doses of 10 mg/kg body weight in SNU-1066 tumor bearing mice. However, tumor volume was rebound and increased after cetuximab treatment. The relative tumor volumes of saline- and cetuximab-treated group were 4-fold and 2-fold increased, compared with tumor volume before treatment. The cetuximab treatment was well tolerated in SNU-1066 xenograft model. There was no apparent body weight loss (Supplementary Figure 1). These results suggest that SNU-1066 HNSCC model has resistant phenotype to immunotherapy of cetuximab in similar with clinical situation.

### Biodistribution of ^64^Cu- and ^177^Lu-PCTA-cetuximab

**Figure 2:**
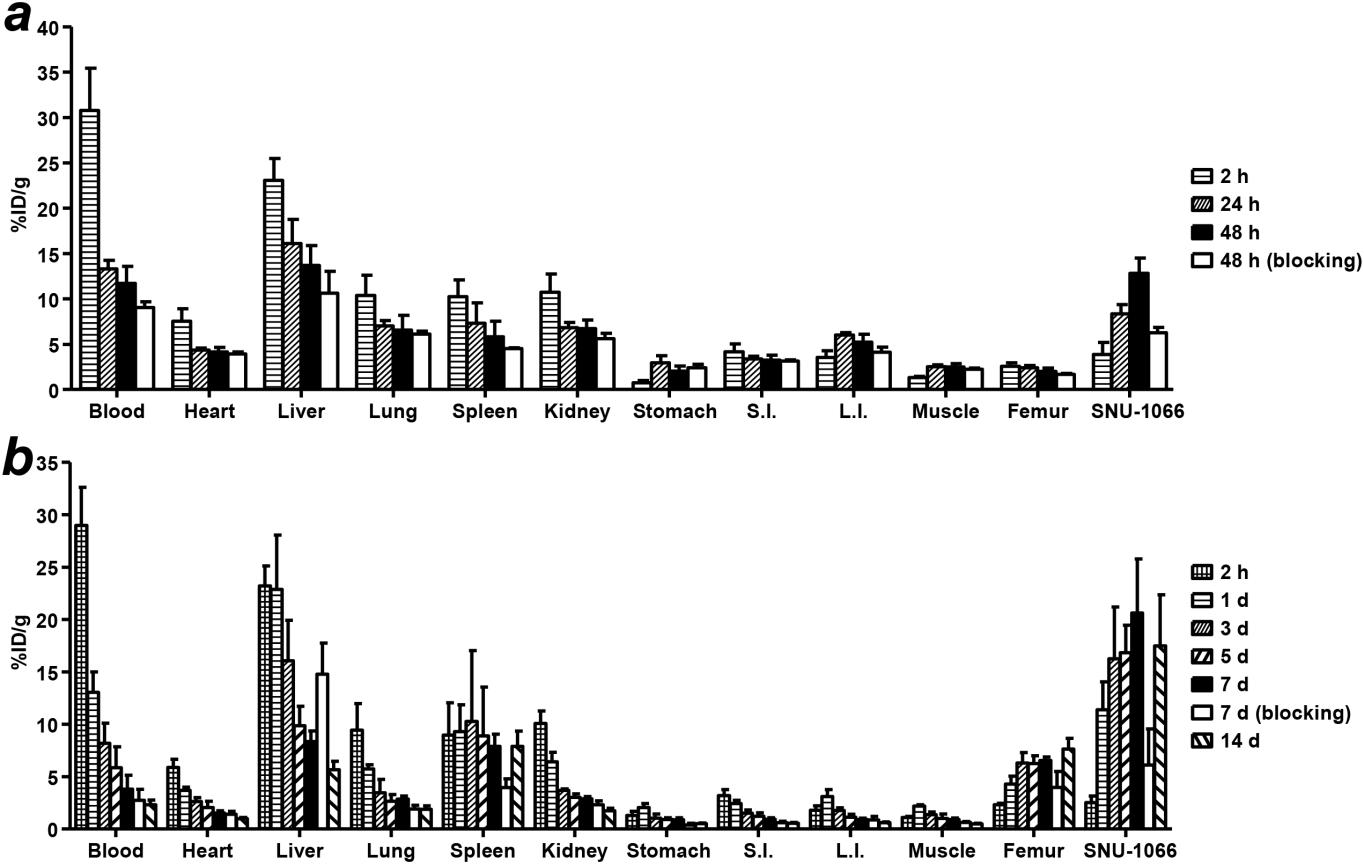
Biodistribution of ^64^Cu-PCTA-cetuximab and ^177^Lu-PCTA-cetuximab in SNU-1066 HNSCC xenograft model ^64^Cu-PCTA-cetuximab **(a)** or ^177^Lu-PCTA-cetuximab **(b)** was injected intravenously into mice. For each time point, the mice were sacrificed and the percentage of the injected radioactivity dose/gram (%ID/g) was determined. In blocking experiments, the uptake of radiolabeled cetuximab in SNU-1066 tumors was significantly reduced by pre-treatment of cold cetuximab. Data were presented as mean ± SD (*n* = 4). S.I., small intestine; L.I., large intestine.

Biodistribution data of ^64^Cu-PCTA-cetuximab at 2, 24, 48 and 72 h taken from the mouse model carrying SNU-1066 tumors were summarized in Figure 2a and Table 1. The radioactivities of the blood and liver were high at 2 h, but gradually decreased over time. The liver uptake of ^64^Cu-PCTA-cetuximab was the highest among normal organs. The SNU-1066 tumor uptake of ^64^Cu-PCTA-cetuximab steadily increased and peaked at 48 h with 12.8 ± 1.7 %ID/g. The tumor-to-blood (T/B) ratios were 0.1 ± 0.0, 0.6 ± 0.1, 1.1 ± 0.1 and the tumor-to-muscle (T/M) ratios were 2.9 ± 0.7, 3.3 ± 0.5, 5.2 ± 0.9 at 2, 24, 48 and 72 h post injection, respectively. In blocking study, the SNU-1066 tumor uptake of ^64^Cu-PCTA-cetuximab significantly reduced to 48.8% at 48 h post-injection, compared to that of ^64^Cu-PCTA-cetuximab without blocking (*P* < 0.01).

**Table 1:** The tumor and backgrounds uptake and Tumor-to-background ratios of ^64^Cu-PCTA-cetuximab in SNU-1066 xenograft model

	2 h			24 h			48 h		
Tumor	3.9	±	1.3	8.4	±	1.0	12.8	±	1.7
Blood	30.8	±	4.7	13.3	±	1.0	11.7	±	1.9
Muscle	1.3	±	0.1	2.5	±	0.2	2.5	±	0.4
Tumor/Blood	0.1	±	0.02	0.6	±	0.1	1.1	±	0.1
Tumor/Muscle	2.9	±	0.7	3.3	±	0.5	5.2	±	0.9

Data represent mean ± S.D (*n* = 4).

The biodistribution data of ^177^Lu-PCTA-cetuximab were also obtained in SNU-1066 tumor-bearing mice (Figure 2b and Table 2). The radioactivity in the blood was 29.0 ± 3.6 %ID/g at 2 h, followed by relatively fast clearance by the end of 14 days (2.3 ± 0.4 %ID/g). The radioactivity of ^177^Lu decreased with time in all organs, except for the SNU-1066 tumor which continued to accumulate radioactivity up to 7 days after administration. The maximum tumor uptake was 20.6 ± 5.2 %ID/g on day 7 and then decreased to 17.5 ± 4.9 %ID/g on day 14. The tumor-to-blood (T/B) and tumor-to-muscle (T/M) ratios of ^177^Lu-PCTA-cetuximab were 6.3 ± 3.8 and 21.9 ± 2.7 at 7 day and were highest with 7.7 ± 2.6 and 36.4 ± 13.1 at 14 day post-injection, respectively. Pre-injection of a blocking dose of cetuximab markedly reduced the tumor uptakes of ^177^Lu-PCTA-cetuximab to 29.7% at 7 days post-injection, compared with that of ^177^Lu-PCTA-cetuximab without blocking (*P* < 0.01). These results represent that ^64^Cu- and ^177^Lu-PCTA-cetuximab have good specificity in EGFR expressing HNSCC xenograft model.

**Table 2:** The tumor and backgrounds uptake and tumor-to-background ratios of ^177^Lu-PCTA-cetuximab in SNU-1066 xenograft model

	2 h			1 day			3 day			5 day			7 day			14 day		
Tumor	2.5	±	0.6	11.4	±	2.6	17.9	±	5.3	16.8	±	2.6	20.6	±	5.2	17.5	±	4.9
Blood	29.0	±	3.6	13.0	±	2.0	8.9	±	2.1	5.8	±	2.0	3.8	±	1.3	2.3	±	0.4
Muscle	1.1	±	0.1	2.2	±	0.1	1.4	±	0.2	1.0	±	0.4	0.9	±	0.1	0.5	±	0.1
Tumor/Blood	0.1	±	0.0	0.9	±	0.1	2.0	±	0.4	3.1	±	1.0	6.3	±	3.8	7.7	±	2.6
Tumor/Muscle	2.3	±	0.7	5.1	±	1.0	12.7	±	2.2	19.4	±	8.2	21.9	±	2.7	36.4	±	13.1

Data represent mean ± S.D (*n* = 4).

### Small animal PET imaging of ^64^Cu-PCTA-cetuximab

**Figure 3:**
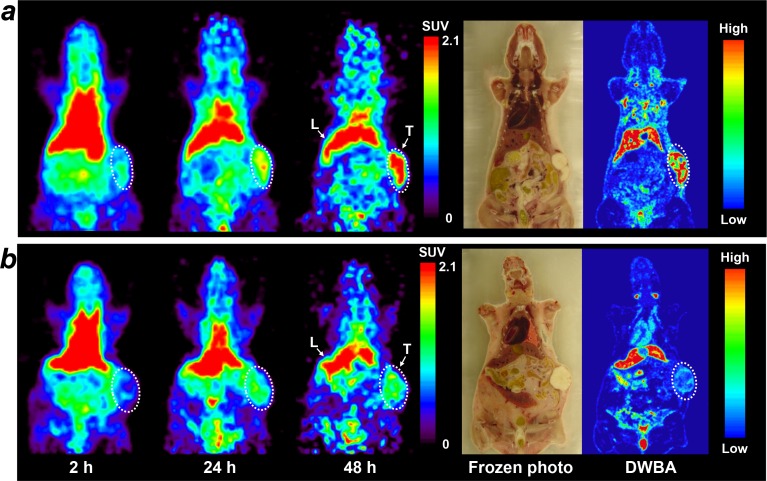
Small animal PET imaging of ^64^Cu-PCTA-cetuximab in SNU-1066 HNSCC xenograft model PET images were acquired at 2 h, 24 h and 48 h after injection of 64Cu-PCTA-cetuximab and represented as SUV. **(a)** ^64^Cu-PCTA-cetuximab was selectively localized in SNU-1066 tumor. After PET imaging, mice were immediately frozen and sectioned, and frozen section photo and digital whole body autoradiography (DWBA) images were obtained. **(b)** In blocking experiments, ^64^Cu-PCTA-cetuximab uptake in tumor was markedly reduced by pre-treated cold excess cetuximab. Tumors are indicated by white dotted circles. T, SNU-1066 tumor; L, liver.

Small-animal PET imaging performed to evaluate the potential of ^64^Cu-PCTA-cetuximab as an immuno-PET imaging agent for EGFR expression level in SNU-1066 tumor-bearing mice (Figure 3). SNU-1066 tumors were clearly visualized on PET images and the tumor uptake of ^64^Cu-PCTA-cetuximab peaked at 48 h post-injection. Physiological liver uptake was also observed, but gradually reduced as a time dependent manner. The tumor SUV of ^64^Cu-cetuximab was 0.9 ± 0.2, 1.9 ± 0.3 and 3.0 ± 0.7 at 2, 24 and 48 h post-injection, respectively. Immuno-PET images were well consistent with the biodistribution data. Blocking experiment with excess dose of cetuximab resulted in 56.7% reduced tumor uptake of ^64^Cu-PCTA-cetuximab, indicating the EGFR targeting specificity of ^64^Cu-PCTA-cetuximab. Digital whole body autoradiography (DWBA) images showed a similar distribution pattern with the PET images.

### Micro-SPECT/CT imaging of ^177^Lu-PCTA-cetuximab

**Figure 4:**
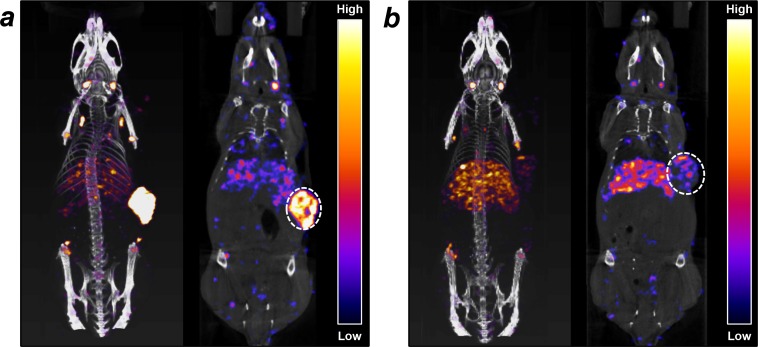
Micro-SPECT/CT images of ^177^Lu-PCTA-cetuximab in SNU-1066 HNSCC xenograft model **(a)** At 7 days post-injection of ^177^Lu-PCTA-cetuximab (12.95 MBq/100 μg), SPECT/CT volume and coronal section images were acquired for 2 h with small animal SPECT/CT system (Bioscan). **(b)** In blocking experiment, SPECT/CT volume and coronal section images were acquired by 2 h pre-injection of excess cetuximab before administration of ^177^Lu-PCTA-cetuximab. Tumors are indicated by white dotted circles.

Micro-SPECT/CT imaging performed to investigate *in vivo* behavior of ^177^Lu-PCTA-cetuximab. Representative SPECT/CT volume images and coronal images of SNU-1066 tumor-bearing mice at 7 day after injection of ^177^Lu-PCTA-cetuximab were shown in Figure 4. ^177^Lu-PCTA-cetuximab was selectively localized in SNU-1066 tumor and showed relatively low uptake in the liver. In the blocking experiments, the tumor uptake of ^177^Lu-PCTA-cetuximab was markedly reduced by administration of excess cold cetuximab, indicating that ^177^Lu-PCTA-cetuximab was specifically localized in EGFR expressing SNU-1066 HNSCC xenograft.

### Radioimmunotherapy

**Figure 5:**
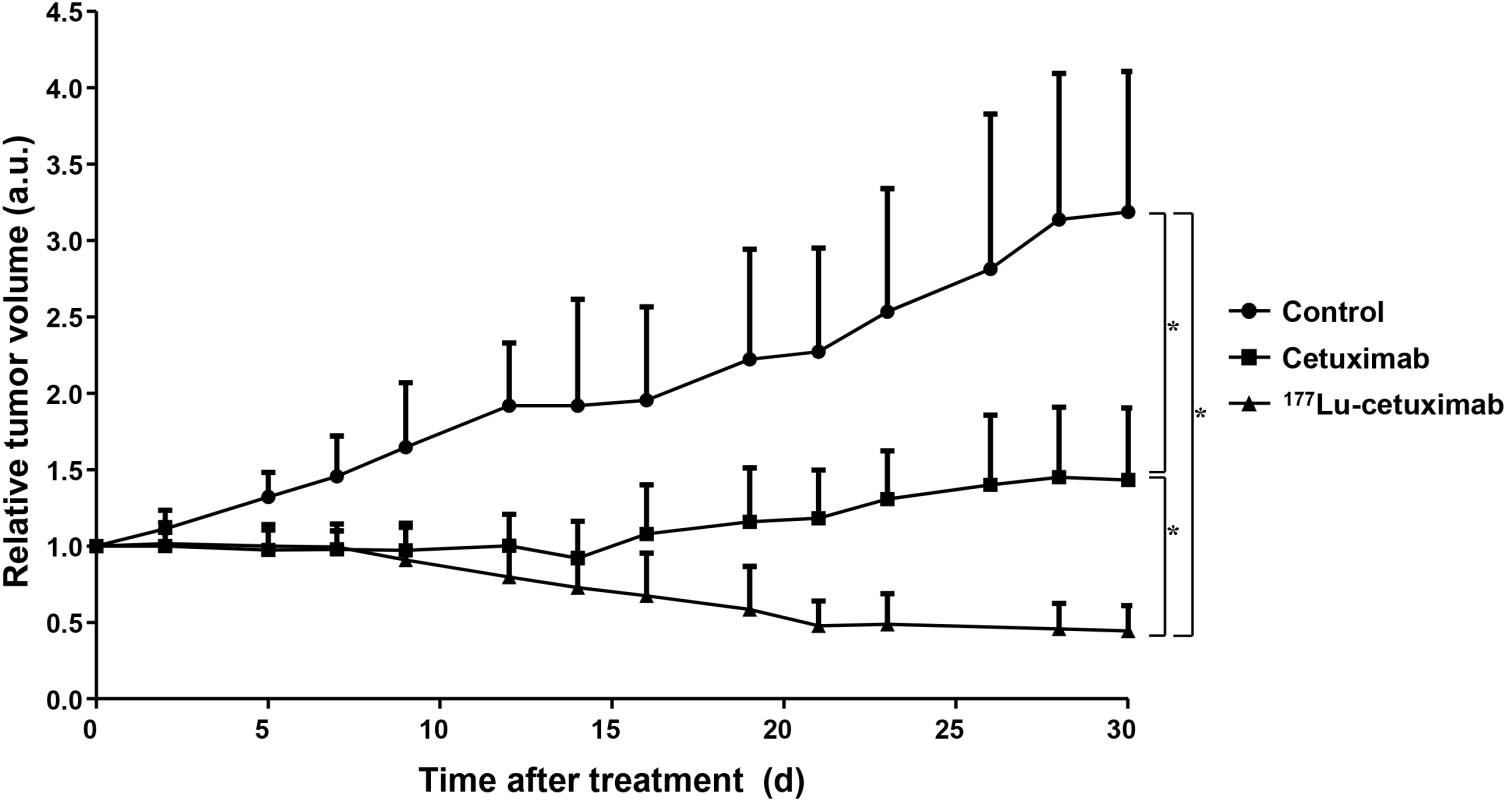
Radioimmunotherpeutic efficacy of ^177^Lu-PCTA-cetuximab in SNU-1066 HNSCC xenograft model The treatment was initiated on day 0. SNU-1066 tumor-bearing mice were treated with saline, cetuximab (single dose), and ^177^Lu-PCTA-cetuximab (12.95 MBq/100 μg). Tumor volume was calculated by caliper measurement. SNU-1066 tumor growth was significantly regressed by ^177^Lu-PCTA-cetuximab treatment. Cetuximab treated group maintained the growth of SNU-1066 tumors for 2 weeks, however, tumor volume re-increased. Data represents mean relative tumor volumes ± SD. ^*^, *P* < 0.05; a.u., arbitrary unit.

We investigated the *in vivo* therapeutic efficacy of ^177^Lu-PCTA-cetuximab RIT in SNU-1066 tumor model (Figure 5). A time-dependent increase in tumor volume was observed in the saline-treated group. A single dose of cetuximab treatment slightly delayed or inhibited the tumor growth during treatment. However, tumor regrowth was observed in cetuximab-treated groups and the average tumor volume increased until 30 day. By contrast, a single-dose injection (12.95 MBq) of ^177^Lu-PCTA-cetuximab treatment showed marked regression of tumor volume. The tumor volume in ^177^Lu-PCTA-cetuximab-treated group on day 30 showed a 55% reduction compared with tumor volume before treatment. The tumor volume in ^177^Lu-PCTA-cetuximab-treated group showed a statistically significant difference compared with that in saline- and single dose of cetuximab-treated groups (*P* < 0.05). SNU-1066 tumor models were well tolerated by ^177^Lu-PCTA-cetuximab treatment, and no apparent body weight loss was observed (Supplementary Figure 2). These results suggest that the 12.95 MBq dose used in this study had no observable toxicity on mice.

### Radiation dosimetry of ^177^Lu-PCTA-cetuximab

For the clinical translation of ^177^Lu-PCTA-cetuximab as a radioimmunotherapeutic agent, human dosimetry is important. The absorbed doses for the major organs and whole body were estimated by the biodistribution data in SNU-1066 tumor-bearing mice are presented in Table 3. Lower large intestine, liver, lung, and spleen showed relatively higher radiation absorbed doses. The whole body absorbed dose was determined to be 0.39 mGy/MBq administrated. However, the radiation dose of red marrow was relatively low. The tumor radiation dose in SNU-1066 HNSCC xenograft model was estimated by a sphere tumor model. Tumor mass was 0.15 ± 0.4 g in RIT treatment group. Absorbed radiation dose of SNU-1066 HNSCC tumor was 67.2 ± 14.6 Gy/ 12.95 MBq of RIT dose.

**Table 3:** Extrapolated radiation dosimetry to an adult human after intravenous injection of ^177^Lu-PCTA-cetuximab based on the biodistribution data obtained in SNU-1066 xenograft model

Organ	mGy/MBq			rad/mCi		
Adrenals	7.3E-05	±	4.6E-06	2.7E-04	±	1.7E-05
Brain	2.9E-07	±	1.8E-08	1.1E-06	±	6.6E-08
Breasts	2.9E-04	±	1.5E-05	1.1E-03	±	5.5E-05
LLI ^*^	2.8E-03	±	1.8E-03	1.0E-02	±	6.6E-03
Small Intestine	5.5E-04	±	3.5E-04	2.0E-03	±	1.3E-03
Stomach	3.7E-03	±	8.6E-04	1.4E-02	±	3.2E-03
ULI ^**^	2.9E-05	±	5.1E-06	1.1E-04	±	1.9E-05
Kidneys	5.7E-03	±	3.1E-04	2.1E-02	±	1.2E-03
Liver	6.0E-02	±	4.5E-03	2.2E-01	±	1.7E-02
Lungs	7.7E-02	±	6.1E-03	2.9E-01	±	2.3E-02
Muscle	1.6E-05	±	1.2E-06	5.8E-05	±	4.4E-06
Ovaries	8.6E-04	±	2.8E-04	3.2E-03	±	1.0E-03
Pancreas	1.1E-04	±	9.6E-06	3.9E-04	±	3.6E-05
Red Marrow	8.2E-04	±	6.5E-05	3.0E-03	±	2.4E-04
Osteogenic Cells	8.3E-05	±	5.8E-06	3.1E-04	±	2.2E-05
Skin	2.5E-05	±	1.7E-06	9.3E-05	±	6.2E-06
Spleen	2.3E-01	±	3.1E-02	8.7E-01	±	1.2E-01
Thymus	1.9E-05	±	7.9E-07	7.2E-05	±	2.9E-06
Thyroid	6.3E-05	±	3.8E-06	2.3E-04	±	1.4E-05
Urinary Bladder	5.8E-05	±	1.6E-05	2.1E-04	±	5.9E-05
Uterus	9.2E-06	±	3.0E-06	3.4E-05	±	1.1E-05
Whole body	3.9E-01			1.4E+00		

^*^ LLI; lower large intestine, ^**^; upper large intestine.

### Therapeutic response monitoring by ^18^F-FDG-PET

**Figure 6:**
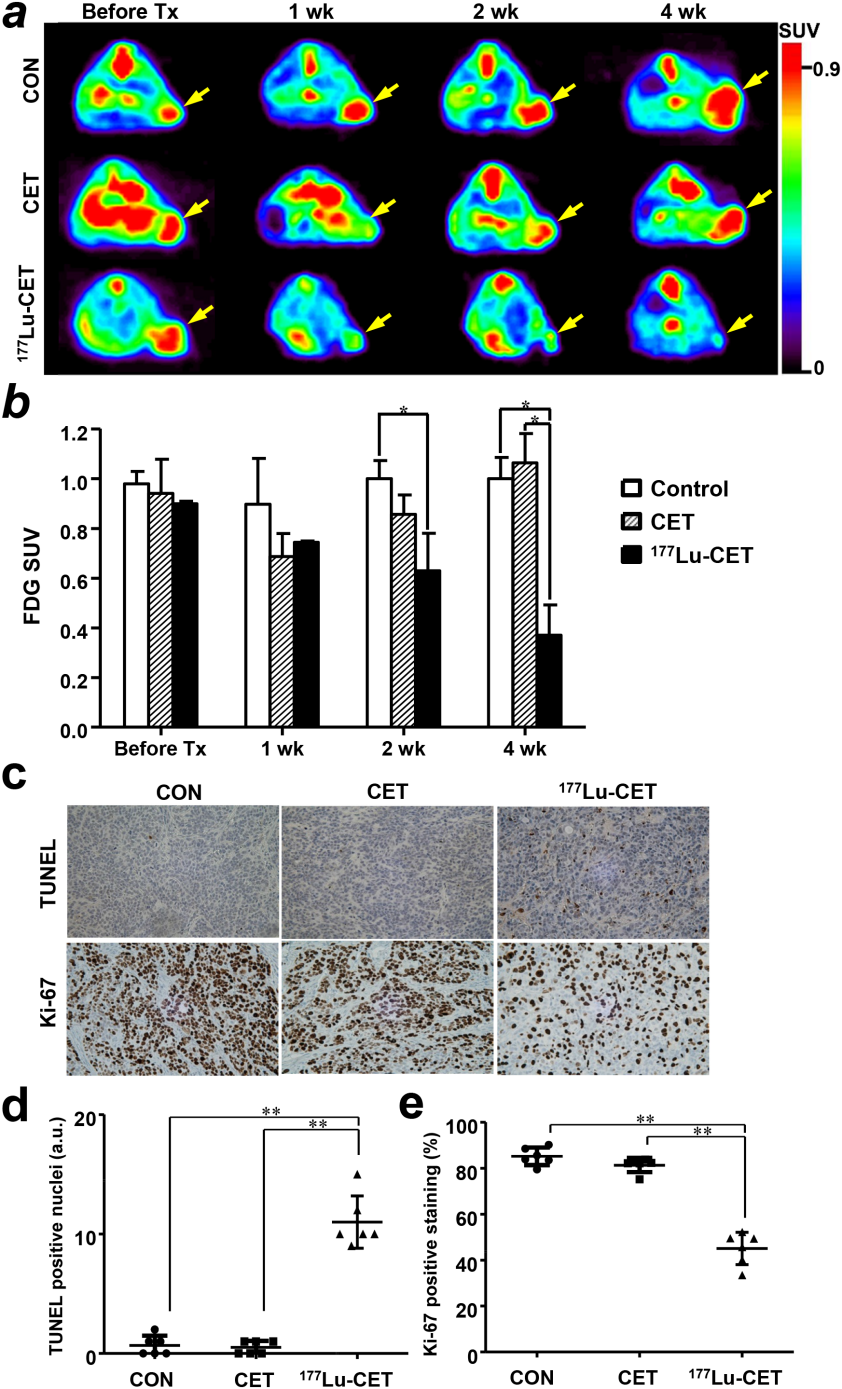
Therapeutic response monitoring with 18F-FDG-PET imaging and immunohistochemical staining **(a)** Glucose metabolic activity was measured by ^18^F-FDG-PET imaging in SNU-1066 xenograft model. ^18^F-FDG-PET images were obtained before treatment and at 1, 2, and 4 weeks after treatment with saline, cetuximab, or ^177^Lu-PCTA-cetuximab. PET images represented as SUV and SNU-1066 tumors are indicated by yellow arrows. **(b)** ^18^F-FDG uptake in tumors was quantified from small animal PET images (*n* = 3). ^177^Lu-PCTA-cetuximab treatment group showed a marked reduction in FDG uptake compared with other groups. ^*^; *P* < 0.05. (**c, d** and **e**) In TUNEL staining in SNU-1066 tumors, there were statistically significant differences in apoptosis level among ^177^Lu-PCTA-cetuximab, saline and cetuximab treatment groups. Ki-67 positive cells in SNU-1066 tumors were markedly reduced in ^177^Lu-PCTA-cetuximab treatment group, in comparison with the saline and cetuximab treatment groups. ^**^, *P* < 0.01. ×400 magnification.

There was little difference in ^18^F-FDG uptake of the saline-treated group for 4 weeks. In single dose of cetuximab treatment group, ^18^F-FDG SUV was reduced by immunotherapy at 1 week. However, SUV re-increased by 2 weeks, and was fully restored to the level of SUV before treatment at 4 weeks. In contrast, the ^177^Lu-PCTA-cetuximab treatment group (0.37 ± 0.12) showed marked reduction of ^18^F-FDG SUV compared to the saline (1.00 ± 0.08) or cetuximab treatment (1.06 ± 0.12) groups at 4 weeks after treatment (*P* < 0.05) (Figure 6a and 6b).

### Immunohistochemistry

To determine the mechanism by which ^177^Lu-PCTA-cetuximab inhibited tumor growth in nude mice, we determined apoptosis and proliferation index using TUNEL and Ki-67 immunohistochemical staining on 14 day after treatment. There were statistically significant differences of apoptotic index in the ^177^Lu-PCTA-cetuximab (14.5 ± 7.0 cells per field) treated group compared to saline (0.7 ± 0.8) and cetuximab (0.5 ± 0.5) treated groups (*P* < 0.01; Figure 6c and 6d), suggesting that the reduction of tumor volume was caused by apoptosis. Ki-67 positive staining (%) were noticeably reduced in the ^177^Lu-PCTA-cetuximab treatment group. The number of Ki-67 positive cell in the ^177^Lu-PCTA-cetuximab treatment group significantly lower than in other groups (saline: 85.2 ± 3.8 vs. cetuximab: 81.3 ± 3.1 vs. ^177^Lu-PCTA-cetuximab: 45.1 ± 7.0, *P* < 0.01; Figure 6c and 6e). Cetuximab treatment group showed slightly reduced the percentage of Ki-67 positive staining. However, it was not statistically significant.

## DISCUSSION

Cetuximab is considered as a promising therapeutic agent in EGFR overexpressed and/or abnormally activated tumors including HNSCC, metastatic colorectal cancer and non-small cell lung cancer [26–28]. Cetuximab has been shown to inhibit the proliferation of various EGFR over-expressed malignant cell lines *in vitro* and enhance the anti-tumor activity of several chemotherapeutic drugs or radiotherapy in mouse xenograft models [29–31]. Although cetuximab treatment has yielded clinical benefit, both intrinsic and acquired resistance are common clinical outcomes. Alternative approach modifying anti-EGFR antibody to deliver cytotoxic molecules including toxins, cytokines, or radioisotopes could be used to overcome the resistance against anti-EGFR antibody treatment. Several previous papers present that therapeutic radionuclides, ^90^Y- and ^177^Lu-labeled anti-EGFR antibodies have the therapeutic potential to cetuximab-resistant HNSCC tumor model [19, 20]. In this study, we performed immuno-PET imaging and radioimmunotherapy using diagnostic and therapeutic convergence radiopharmaceutical, ^64^Cu/^177^Lu-PCTA-cetuximab in cetuximab-resistant SNU-1066 HNSCC xenograft model.

We identified that SNU-1066 HNSCC tumor has the characteristics of clinical-mimicking cetuximab-resistant phenotype. *In vitro* cytotoxicity test showed that cetuximab treatment did not have significant therapeutic effect (Figure 1c). In SNU-1066 xenograft model, six doses of cetuximab immunotherapy for 2 weeks did not have good therapeutic efficacy, which tumor volumes sustained during treatment and increased after treatment (Figure 1f). However, single dose of radioimmunotherapy with ^177^Lu-PCTA-cetuximab showed greater therapeutic effect than cetuximab immunotherapy without significant toxicity (Figure 5 and supplementary Figure 2). We think that the therapeutic effect of ^177^Lu-PCTA-cetuximab mainly resulted from beta-irradiation of ^177^Lu delivered by cetuximab.

In HNSCC cells binding assay, ^64^Cu-PCTA-cetuximab demonstrated that cell bound radioactivity (%) was well correlated with the EGFR level of HNSCC cells evaluated by flow cytometry and western blot analysis (Figure 1a, 1b, and 1d). ^64^Cu-PCTA-cetuximab was selectively accumulated in SNU-1066 HNSCC tumors and showed good tumor-to-background ratios in immuno-PET imaging and biodistribution study (Figure 2 and 3). In blocking study, the tumor uptake of ^64^Cu-PCTA-cetuximab was markedly reduced in SNU-1066 xenograft models, which represent the specificity of EGFR targeting of ^64^Cu-PCTA-cetuximab. In clinical situation, ^64^Cu-PCTA-cetuximab immuno-PET imaging could be a useful surrogate imaging biomarker that quantitatively evaluate the EGFR expression level and select pertinent HNSCC patients for radioimmunotherapy in cetuximab-resistant HNSCC patients.

Immuno-PET is attractive for studying the *in vivo* behavior of therapeutic antibodies and their interaction with critical disease targets, because it enables quantitative imaging of antibodies at high resolution and sensitivity. Immuno-PET imaging has been extensively studied through optimal combination between various antibodies and genetically engineered forms (intact IgG, ScFv, minibody, diabody, ScFv, and affibody, et al.) and positon emitting radionuclides (^68^Ga, ^18^F, ^64^Cu, ^86^Y, ^89^Zr, ^124^I) on the basis of the pharmacokinetic parameters of antibodies and its derivatives and the pharmacodynamics of target molecules [32–34]. As most therapeutic antibodies are intact IgG form, immuno-PET also mainly focused on the whole IgG antibodies and the associated use of the long-lived positron emitter, ^89^Zr. Immuno-PET with ^89^Zr-labeled antibodies has been studied for their targeting characteristics in clinical patients. Immuno-PET imaging seems to be safe, because diagnostic method needs repetitive imaging. In dosimetry study, immuno-PET with ^89^Zr-labeled antibodies usually 8-times higher whole body effective dose (40 mSv/74 MBq in ^89^Zr vs. 5 mSv/130 MBq in ^64^Cu) than that with ^64^Cu-labeled antibodies in clinical patients [23]. In aspect of dosimetry, immuno-PET with ^64^Cu-labeled antibodies has favorable parameters. With immuno-PET, the presence and extent of desired target in primary and metastatic lesions can be demonstrated noninvasively in whole-body imaging early in clinical development such as *in vivo* immunohistochemistry. This also allow broad potential application in cancer detection and staging, tumor and metastasis phenotyping, stratification of patients into treatment groups, and evaluation of tumor targeting and therapy response and provide useful comprehensive information for optimizing doses of radioimmunotherapy, for preventing adverse effects, and for thus contributing one of the personalized precision medicine [23, 33].

The therapeutic effect of RIT mainly depends on the target accumulation and radiation dosimetry that is delivered to targets. Our study determined whole body radiation dosimetry of ^177^Lu-PCTA-cetuximab through extrapolation of biodistribution data from mice. ^177^Lu-PCTA-cetuximab showed favorable whole body effective radiation dose (Table 3). In conventional radiation therapy, high radiation doses are needed to achieve clinical responses in solid tumors such as glioma (60 Gy) [35], breast cancer (50 Gy) [36], and pancreatic cancer (50.4 Gy) [37]. Based on these clinical experiences, it is generally accepted that solid tumor doses need to reach at least 50 Gy to achieve any clinical benefits. Unfortunately, tumor radiation doses are below 50 Gy in previously reported radioimmunotherapy studies of solid tumors. In a phase I trial of ^131^I labeled anti-TAG72 antibody (CC49) to treat metastatic gastrointestinal cancers, tumor absorbed doses in metastatic sites ranged from 6.3 to 33 Gy [38]. In a similar study, the tumor doses for ^90^Y-CC49 were found to be 1.8 to 30 Gy [39]. Our results showed that tumor radiation dose of ^177^Lu-PCTA-cetuximab in SNU-1066 tumor was above 60 Gy, in case of 12.95 MBq of ^177^Lu-PCTA-cetuximab treatment, deduced from tumor mass of RIT treatment group. This radiation dose is thought to be sufficient to achieve clinical benefits in the tumor while maintaining low dose exposure in other organs.

Immunotherapy using tumor-targeted antibodies has experienced problems such as rapid tumor regrowth or the “rebound” radiographic phenomenon after cessation of maintenance treatment. We observed tumor regrowth over time in single-dose (100 μg) and multiple-dose (200 μg, 6 times/2 weeks) cetuximab treatments (Figure 1f and 5). However, there was no tumor regrowth or rebound phenomenon in ^177^Lu-PCTA-cetuximab treatment group. These data suggest that the radiation effect by beta emitting radioisotopes inhibit the tumor regrowth or rebound phenomena. ^177^Lu displays low-energy β^−^-emission (497 keV, 78.7%) with minimal tissue penetration, making it suitable for therapy of small and metastatic tumors [24]. In the present study, we focused on evaluating improved therapeutic effects of RIT using ^177^Lu-PCTA-cetuximab compared to cetuximab-based immunotherapy alone. In future studies, the therapeutic efficacy of RIT for EGFR expressing HNSCC tumors that are resistant or less responsive to cetuximab or the combination therapeutic effect with cisplatin will be investigated in various HNSCC xenograft models.

^18^F-FDG PET imaging has been used for tumor volume assessment and staging and known as a good predictive marker of cetuximab immunotherapy and investigated as a potential early biomarker of cetuximab therapeutic effect in HNSCC patients [40]. In our study, single dose of cetuximab treatment in SNU-1066 tumor showed reduced ^18^F-FDG tumor uptake at 1 week, but their tumor uptake was rebound to that of control tumors. In radioimmunotherapy, ^18^F-FDG tumor uptake consistently decreased to 40% of the tumor uptake before RIT treatment and in saline treated group (Figure 6a and 6b). We suggest that ^18^F-FDG-PET imaging could be used for stratifying patients with cetuximab-resistant HNSCC tumor and monitoring therapeutic efficacy of RIT.

In conclusion, we described the feasibility of immuno-PET imaging based radioimmunotherapy with a diagnostic and therapeutic convergence radiopharmaceutical, ^64^Cu/^177^Lu-PCTA-cetuximab in cetuximab-resistant SNU-1066 HNSCC xenograft model. Immuno-PET with ^64^Cu-PCTA-cetuximab exhibited good tumor targeting and target-to-background ratio. Radioimmunotherapy with ^177^Lu-PCTA-cetuximab showed significant therapeutic efficacy in cetuximab-resistant SNU-1066 HNSCC tumors. The convergence radiopharmaceutical, ^64^Cu/^177^Lu-PCTA-cetuximab for immuno-PET imaging and radioimmunotherapeutic agent may provide a personalized regimen that can be individualized each patient through the quantitative visualization of target molecules by immuno-PET imaging as well as selective treatment by radioimmunotherapy.

## MATERIALS AND METHODS

### Cell culture

Human head and neck squamous cell carcinoma YD-8, SNU-1041, SNU-1066, and SNU-1076 cells were purchased from Korean Cell Line Bank (Seoul, South Korea). YD-8, SNU-1041, SNU-1066, and SNU-1076 cells were grown in RPMI 1640 (Invitrogen, Carlsbad, CA, USA) containing 10% fetal bovine serum (FBS; Hyclone) at 37°C in 5% CO_2_ humidified incubator.

### Western blot analysis

HNSCC cells (YD-8, SNU-1041, SNU-1066, SNU-1076) were lysed in RIPA buffer (Thermo Scientific, Rockford, IL, USA) containing protease inhibitors (GenDEPOT, Katy, TX, USA) for protein extraction. Western blot was performed according to standard protocols (Bio-RAD Laboratories, Inc., Hercules, CA, USA). Protein lysates (25 μg) were separated using 10% SDS-polyacrylamide gels and transferred to nitrocellulose blotting membranes (GE Healthcare Life Sciences, Chicago, IL, USA). After gel transfer, the membrane was incubated with blocking solution, agitated for 1 h at room temperature, and then probed with anti-EGFR primary (Santa Cruz Biotechnology, Santa Cruz, CA, USA) and appropriate secondary antibodies. After three additional washes in TBS-T, specific proteins were detected using a chemiluminescence detection system. β-actin was used as a loading control.

### Flow cytometry

HNSCC cells were harvested and washed with PBS containing 3% bovine serum albumin. The cells were incubated with cetuximab (10 μg) for 1 h. The isotype control group was incubated with rituximab (10 μg) for 1 h. After the washes, monoclonal anti-human FITC-conjugated IgG antibody (Sigma-Aldrich, St. Louis, MO, USA) was added to cells and incubated for 1 h at 4°C. The cells were washed three times with PBS and analyzed using FACS Calibur (Becton Dickinson Biosciences, Franklin Lakes, NJ, USA) to measure the expression level of EGFR at the cell surface.

### Cell viability assay

Cell viability was determined using MTS assay. SNU-1066 cells were seeded in 96-well plates at a density of 4 × 10^3^ cells/well. After overnight incubation, the media was removed and cells were treated with different doses of cetuximab (0, 1, 10, 50, 100 μg/mL) for 3 and 5 days. The cells were then incubated with Celltiter 96 AQueous One (MTS) solution (Promega, Madison, WI, USA) for 1 h. Absorbance was measured at 490 nm using a microplate reader (BioTek, Winooski, VT, USA).

### Preparation of PCTA-cetuximab immunoconjugate

Immunoconjugates were prepared by previous protocol [25]. Briefly, cetuximab (20 mg) reacted with the bi-functional chelator, *p*-SCN-Bn-PCTA (Macrocyclics, Dallas, TX, USA) (10 equivalents) in 100 mM sodium bicarbonate buffer, pH 8.5 at room temp for 2 h and continued at 4°C overnight. Unconjugated chelator was removed by dialysis. The immunoconjugate was finally concentrated to 2 mg/mL in 20 mM sodium acetate buffer, pH 6.5. To determine the number of chelates per antibody, mass spectrometry was performed by using MALDI mass spectrometry (Voyager-DE STR, PerSpective Biosystems Inc., KBSI, Ohchang, Republic of Korea).

### Radiolabeling

^64^Cu was produced by 50 MeV cyclotron irradiation at KIRAMS. ^177^Lu was purchased from ITM AG. ^64^CuCl_2_ (74 MBq) or ^177^LuCl_3_ (74∼740 MBq) was added to 1 mg of PCTA-cetuximab. The reaction mixtures were incubated for 1 h at room temperature or 37°C, respectively, with constant shaking. Radiolabeling yield and purity were assessed by instant thin-layer chromatography silica gel (Pall Corp.) as the stationary phase and 20 mM citrate buffer, pH 5, with 50 mM EDTA as the mobile phase. Radiochemical purity was also confirmed by size-exclusion high-performance liquid chromatography.

### Cell binding assay

To evaluate the immunoreactivity of ^64^Cu- or ^177^Lu-radiolabeled cetuximab towards EGFR as well as EGFR expression levels in HNSCC cell lines, *in vitro* cell binding assay was done. Cell binding studies with ^64^Cu-/^177^Lu-radiolabeled cetuximab (3.7 kBq/100 ng/tube) were performed using YD-8, SNU-1041, SNU-1066, and SNU-1076 cells (1 × 10^6^ cells/tube, triplicate). Nonspecific binding was determined in the presence of 100-fold-excess of cetuximab. Cell bound radioactivity (%) was calculated using (total cell bound radioactivity - nonspecific binding radioactivity)/total applied radioactivity × 100.

### Cytotoxicity assay of ^177^Lu -PCTA-cetuximab

SNU-1066 cells (5 × 10^4^ cells per well) were seeded in 6-well plates and incubated for 18 h. The cells were treated with ^177^Lu-PCTA-cetuximab at 0.037, 0.37, 0.74, and 1.48 MBq/well in culture media containing 1% FBS. Corresponding control wells were treated with media alone and unlabeled cetuximab in equal concentrations (2 μg/well) to radiolabeled antibody. After 1 h incubation, the treated media was aspirated and fresh media was added to each well. On day 3, 4, and 5 after treatment, the cytotoxic effects of ^177^Lu-PCTA-cetuximab were evaluated by determining cell viability using an automated cell counter (ADAM, Digital-Bio, Seoul, Republic of Korea).

### Immunotherapy

All animal studies were conducted in accordance with guidelines of the Institutional Animal Care and Use committee (IACUC) at the Korea Institute of Radiological and Medical Sciences (KIRAMS). Female athymic mice (6 weeks) were obtained from Nara Biotech (Seoul, Republic of Korea). SNU-1066 cells (1.2 × 10^7^ cells in PBS, pH 7.4) were subcutaneously injected into the right flank of mice. When tumor volume reached 100-200 mm^3^, cetuximab immunotherapy (*n* = 6 ∼ 7/group) was performed. SNU-1066 tumor-bearing mice were treated with saline or cetuximab (10 mg/kg, thrice per week) for 2 weeks. Tumor volume was calculated by (long diameter × short diameter^2^)/2 and data represented as relative tumor volume. Body weight was measured three times a week. Tumor volume and body weight were measured for 42 day.

### Biodistribution study

SNU-1066 cells (1.2 × 10^7^ cells) were subcutaneously injected into the right flank of mice. When tumor volume reached 100-200 mm^3^, biodistribution experiments were performed. Mice bearing SNU-1066 HNSCC tumors were intravenously injected with a 3.7 MBq (100 μg) of ^64^Cu-PCTA-cetuximab or ^177^Lu-PCTA-cetuximab (*n* = 4) and sacrificed at each time point. For the blocking experiment, tumor-bearing mice were pre-injected with 100 mg/kg of cetuximab 2 h prior. The blood, various tissues and SNU-1066 tumors were excised and weighed. The radioactivity was measured using a NaI crystal well-type gamma counter (Wizard 1480, Perkin-Elmer, Waltham, MA, USA) applying a decay correction. Counts were compared with those of standards, and the data were expressed as the percentage of injected radioactivity dose per gram of tissue (%ID/g).

### Immuno-PET imaging

To evaluate tumor targeting of ^64^Cu-PCTA-cetuximab, immuno-PET imaging was performed in SNU-1066 tumor–bearing mice. ^64^Cu-PCTA-cetuximab (3.7 MBq) was intravenously injected into the mice, and static scans were acquired for 60 min at 2, 24, 48, and 72 h after injection using a small animal PET scanner (microPET R4; Concorde Microsystems, Knoxville, TN, USA). To evaluate the specificity of EGFR expressing tumor targeting of ^64^Cu-PCTA-cetuximab, excess cold cetuximab (2 mg/head) was intravenously injected for blocking experiments. Quantitative data were expressed as standard uptake value (SUV) [41]. Images were visualized using ASIPro display software. After PET scanning, mice were immediately euthanized and frozen, and digital whole body autoradiography (DWBA) was performed.

### Micro-SPECT/CT imaging

Micro-SPECT/CT was performed on 7 days after injection of ^177^Lu-PCTA-cetuximab (12.95 MBq, 100 μg) in the SNU-1066 tumor model. To evaluate the specificity of EGFR expressing tumor targeting of ^177^Lu-PCTA-cetuximab, excess cold cetuximab (2 mg/head) was intravenously injected for blocking experiments. Micro-SPECT/CT imaging was performed using a NanoSPECT/CT tomograph (Bioscan, Poway, CA, USA) for 120 min acquisition. Cone-beam CT images were acquired (180 projections, 1 s/projection, 45 kVp, 177 μA) before micro-SPECT imaging. Co-registration of micro-SPECT and CT images was performed using InVivoScope software (ver. 2.0, Bioscan).

### Radioimmunotherapy

SNU-1066 cells were subcutaneously injected into the right flank of mice. When tumor volume reached 100-200 mm^3^, radioimmunotherapy with ^177^Lu-PCTA-cetuximab were performed. SNU-1066 tumor-bearing mice were randomly divided into three groups (*n* = 6 or 7 per group). HNSCC tumor mice were intravenously administrated with saline (control), cetuximab (5 mg/kg, single dose) or ^177^Lu-PCTA-cetuximab (12.95 MBq, single dose, 5 mg/kg), respectively. Tumor volume and body weight were measured for 30 day post-treatment.

### Radiation dosimetry of ^177^Lu-PCTA-cetuximab

Estimated human dosimetry was calculated from the biodistribution results from SNU-1066 tumor-bearing mice injected with about 3.7 MBq of ^177^Lu-PCTA-cetuximab. Time-activity curves were generated from the mean values obtained in mice for each tissue of interest. We then calculated source organ residence times for the human model by integrating a mono-exponential fit to the experimental biodistribution data for the major organs and whole body. Murine normal organ cumulated activities were converted to human normal organ cumulated activities by adjusting for the differences in total body and organ masses between mice and humans (assuming a 70-kg standard human). The calculated human normal organ cumulated activities were entered into the Organ Level Internal Dose Assessment (OLINDA, Vanderbilt University, Nashville, TN, USA) dosimetry computer program v1.0 to calculate, using the formulae of the Medical Internal Dosimetry Committee of the Society of Nuclear Medicine [42], the standard human organ absorbed doses. Extrapolated radiation dosimetry for humans was prepared by assuming that the metabolism rates and pharmacokinetics of ^177^Lu-PCTA-cetuximab in man and mouse are equivalent.

The tumor radiation dose was estimated by previously reported method [43] using a sphere tumor model. The excised tumors from the biodistribution of ^177^Lu-PCTA-cetuximab were round and mostly elliptical, and the assumption of the spherical volume for tumor dosimetry was not far from the actual geometrical shape. Since the OLINDA software only provides the dose estimates (mGy/MBq) discretely for the representative sphere mass in the linear range from 0.01 to 6,000 g, for the specific weight of each tumor, the interpolated value for the dose estimate was obtained using the linear squares fit to logarithmic values of the sphere mass and the radiation dose estimate.

### Therapeutic response monitoring by ^18^F-FDG-PET imaging

To assess metabolic activity to ^177^Lu-PCTA-cetuximab therapy, ^18^F-FDG-PET imaging was performed before treatment and at 1, 2, and 4 weeks after treatment. ^18^F-FDG (7.4 MBq) was injected intravenously 1 h before scanning, and static scans were obtained for 20 min. PET images were analyzed and quantified as described previously [44].

### Immunohistochemistry

Tumor tissues were harvested on day 14 post-treatment and immediately fixed in 4% paraformaldehyde. Apoptosis was detected using a terminal deoxynucleotidyl transferase dUTP nick end-labeling (TUNEL) assay kit (Millipore, Germany) on 4 μm thick sections according to the manufacturer's instructions. Tumor sections were stained with a Ki-67 specific SP6 rabbit mAb (Abcam, UK), and the EnVision detection system for rabbit antibody (Dako, Denmark) was applied according to the manufacturer's instructions. Counterstaining was performed with Mayer's hematoxylin. Nuclear staining of Ki-67 was considered positive. TUNEL-positive nuclei were counted in 6 random fields, and the Ki-67 staining index was defined as the percentage of positive nuclei per 1,000 nuclei.

### Statistical analysis

Quantitative data are represented as the mean ± SD, and statistical analysis was performed by one-way ANOVA or Student's *t* test using Prism 5 (GraphPad Software, La Jolla, CA, USA). *P* values of < 0.05 were considered statistically significant.

## SUPPLEMENTARY MATERIALS FIGURES



## Abbreviations

HNSCC: head and neck squamous cell carcinoma
PET: positron emission tomography
EGFR: epidermal growth factor receptor
RIT: radioimmunotherapy
^18^F-FDG: 2-[^18^F]fluoro-2-deoxy-D-glucose
SPECT/CT: single-photon emission computed tomography/computed tomography
PCTA: 3,6,9,15-tetraazabicyclo[9.3.1]-pentadeca-1(15),11,13-triene-3,6,9,-triacetic acid.
