# Supplementary material for: Immuno-PET imaging based radioimmunotherapy in head and neck squamous cell carcinoma model
Source: Oncotarget. 2017 Sep 8;8(54):92090–105. doi: 10.18632/oncotarget.20760 (PMC5696166; doi:10.18632/oncotarget.20760)
Supplement: Supplementary file 1 [file oncotarget-08-92090-s001.pdf]

Immuno-PET imaging based radioimmunotherapy in head and neck squamous cell carcinoma model

SUPPLEMENTARY MATERIALS

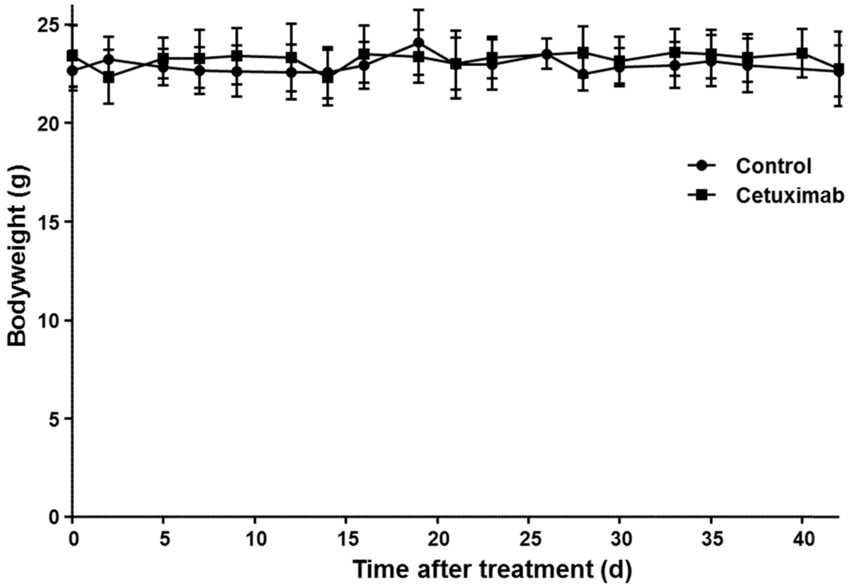

Supplementary Figure 1: Body weight changes in SNU-1066 xenograft model administered saline or cetuximab. Body weight was monitored three times a week during therapy. No significant weight loss was observed in saline (control) and cetuximab treatment groups.

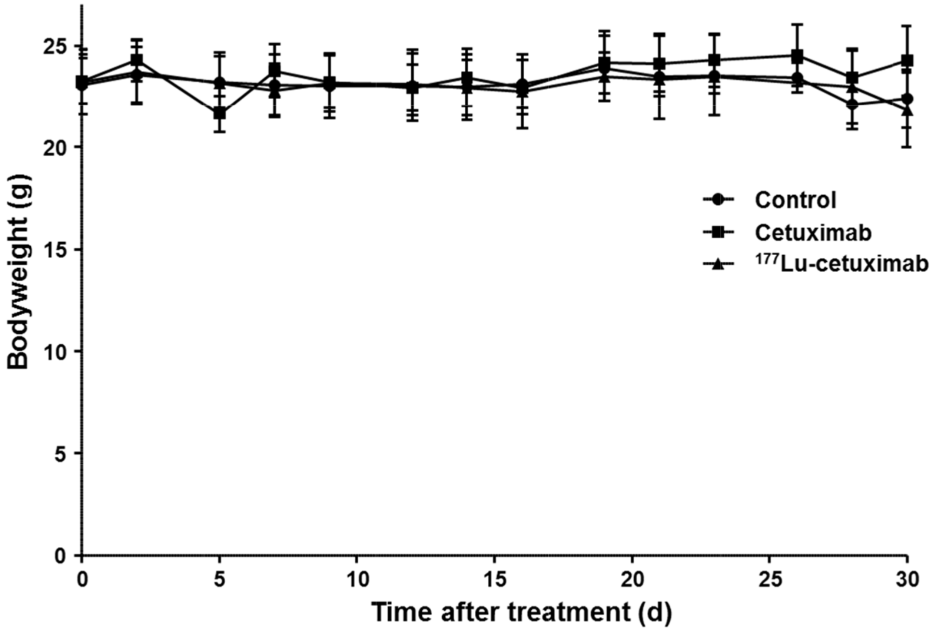

Supplementary Figure 2: Body weight changes in SNU-1066 xenograft model administered saline, cetuximab or ^{177}Lu-PCTA-cetuximab. Body weight was monitored three times a week during therapy. No significant weight loss was observed in saline (control), cetuximab and ^{177}Lu-PCTA-cetuximab treatment groups.
